# Early-Life Events and the Prevalence of Gut–Brain Interaction Disorders in Children

**DOI:** 10.3390/children12111430

**Published:** 2025-10-23

**Authors:** Atchariya Chanpong, Natchayada Ponjorn, Nattaporn Tassanakijpanich, Vanlaya Koosakulchai, Pornruedee Rachatawiriyakul, Sirinthip Kittivisuit, Puttichart Khantee, Kamolwish Laoprasopwattana

**Affiliations:** 1 Division of Gastroenterology and Hepatology, Department of Pediatrics, Faculty of Medicine, Prince of Songkla University, Songkhla 90110, Thailand; atchariya.c@psu.ac.th; 2 Faculty of Medicine, Prince of Songkla University, Songkhla 90110, Thailand; 3 Department of Pediatrics, Faculty of Medicine, Prince of Songkla University, Songkhla 90110, Thailand; nattaporn.t@psu.ac.th (N.T.); vanlaya.koo@gmail.com (V.K.); tingting_chan33@hotmail.com (P.R.); kputtich@gmail.com (P.K.)

**Keywords:** disorders of gut–brain interaction, functional gastrointestinal disorders, breastfeeding, early life, microbiota–gut–brain interaction

## Abstract

**Highlights:**

**What are the main findings?**

• Environmental factors in the first 3 years of life could influence postnatal development and lifelong health and well-being.
• Breastfeeding, particularly for ≥3 months, is the most important protective factor against DGBI, whereas antibiotic/antiviral exposure, particularly in the first year of life, may increase the risk of DGBI development.

**What is the implication of the main finding?**

• Exclusive breastfeeding should be continuously promoted to prevent the occurrence of DGBI.
• The rational use of antibiotics/antivirals should be advocated to decrease the risk of developing DGBI.

**Abstract:**

**Background/Objectives**: Disorders of gut–brain interaction (DGBI) include a spectrum of disorders with chronic/recurrent gastrointestinal symptoms, caused by dysregulation of microbiota–gut–brain interaction. Early-life events have been suggested as the main factors influencing the microbiota–gut–brain axis. We aimed to evaluate the prevalence of DGBI in 3-year-old children and its relationship with early-life events. **Methods**: The parents of children aged 3 years, who had been followed up in a well-baby clinic since they were 2 months old, were asked about any GI symptoms their child had experienced during the check-up visits between September 2023 and June 2024. The final diagnosis of DGBI was based on ROME IV criteria. Demographic data, including early-life factors, were collected. **Results**: Overall, 568 children (48.6% boys) were included, of whom 139 (24.5%) had symptoms consistent with at least one DGBI diagnosis. The most prevalent DGBI was functional constipation (20.4%), followed by colic (4.6%), infant regurgitation (2.8%), and dyschezia (1.6%). Approximately 48% of the children were breastfed for ≥6 months, and 21% were exposed to ≥1 antibiotic/antiviral drugs in the first year of life. DGBI prevalence was significantly higher in girls than in boys (28.1% vs. 20.7%; *p* = 0.041). Exclusive breastfeeding was the most significant protective factor against DGBI, particularly if performed for ≥3 months. **Conclusions**: Sex was the most significant factor affecting DGBI prevalence in children aged ≤3 years; breastfeeding offers the most effective protection against DGBI development.

## 1. Introduction

Early life, or the first 3 years of age, is a period of rapid parallel growth of the gut, brain, and microbiome [1,2]. The developmental interdependence and bidirectional communication between the microbiome, gastrointestinal (GI) tract, and brain forms part of the microbiota–gut–brain axis [1]. Several studies have shown the importance of environmental factors, especially in the early years of life, that influence postnatal development and lifelong health and well-being [2,3,4,5,6,7,8]. Examples of these factors include antibiotic exposure, the delivery method, and breastfeeding [4,5,6,7,8].

During early life, a combination of genetic and medical or psychosocial factors may disturb the structure or function of the microbiota–gut–brain axis [9]. This could lead to disorders of gut–brain interaction (DGBI), previously known as functional gastrointestinal disorders. Medical events potentially leading to DGBI development include dysbiosis from early use of antibiotics or cesarean section and inflammation due to allergy or infection [5,7,10,11,12,13]. Examples of psychosocial factors that could modulate the gut microbiota and brain development include early-life pain, trauma, and stress [14,15,16]. However, most of the supporting evidence on this etiopathogenesis model is based on in vitro and animal studies.

To date, studies on the relationship between early-life events and the prevalence of DGBI are limited, and current findings are conflicting. Hence, we aimed to evaluate the prevalence of DGBI in children aged ≤3 years and identify whether early-life factors, such as the birth method, breastfeeding, antibiotic exposure, and allergic diseases, affect the prevalence of DGBI.

## 2. Materials and Methods

A retrospective–prospective cohort study was performed at Songklanagarind Hospital, Prince of Songkla University in Thailand. All healthy children aged 3 years, who had been followed up with in a well-baby clinic for vaccination since they were 2 months old (as part of a vaccine trial [V114-032; REC 63-380-1-1]), were included in this study. Those who were found to have underlying chronic diseases or malnutrition were excluded.

The sample size was calculated using OpenEpi, Version 3 [17]. Data from Chia et al. [18] were used, who reported a 9.3% prevalence of infant colic with an associated odds ratio (OR) of 0.12 for breastfeeding and a 5.6% prevalence of functional constipation with an OR of 0.06 for breastfeeding. Assuming a confidence level of 95% and power of 80%, a sample size of 240 and 330 children was required to detect the prevalence of colic and functional constipation, respectively. Hence, a total sample size of 570 children was recommended.

**Figure 1:**
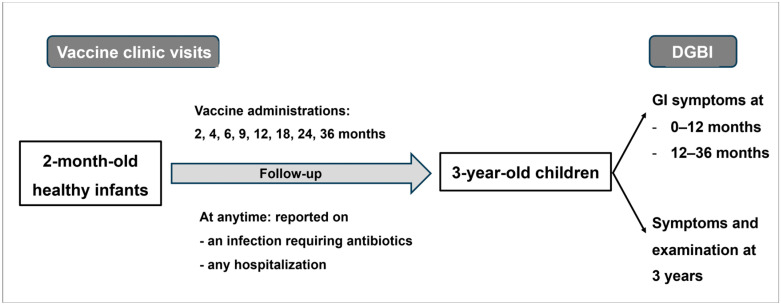
Study flow diagram. DGBI, Disorders of gut–brain interaction.

For the V114-032 study (REC 63-380-1-1), healthy infants were recruited at 2 months of age between October 2020 and August 2023. According to the immunization program, each child was scheduled for vaccine clinic visits every 2 months until 6 months of age, every 3 months until 1 year of age, every 6 months until 2 years of age, and every year thereafter (Figure 1). During the follow-ups, the doctors evaluated the child’s growth and development together with their feeding history. As part of the V114-032 study, pediatric investigators collected data on birth history, infection history during pregnancy, exclusive breastfeeding duration, parental education, family history of atopic diseases, and environment (e.g., secondhand smoking). To determine the age at first antibiotic/antiviral exposure, information was collected between 2 months and 3 years of age. The child’s parents were required to report any illnesses, particularly those requiring hospitalization, and medication use (including dosage and duration). For each report, the child was examined, and their guardians were interviewed before documenting the illness episode and medication use in the system. During the visit at 3 years of age, from September 2023 to June 2024, parents were interviewed by pediatric investigators about any GI symptoms their child had experienced at any time. The final diagnosis of DGBI was made by a pediatric gastroenterologist (Figure 1) based on ROME IV criteria [19]. The investigators evaluated the presence of each condition by asking a set of questions or administering a questionnaire adapted from ROME IV diagnostic criteria. During the first 3 years, the DGBI diagnosis could be infant colic, dyschezia, infantile regurgitation, cyclic vomiting syndrome, functional constipation, or functional diarrhea [19].

The diagnostic criteria for infant colic included all of the following: (1) an infant <5 months of age when the symptoms start and stop; (2) recurrent, prolonged periods (≥3 h/day on ≥3 days in 7) of crying, fussing, or irritability that occur without obvious cause and cannot be prevented or resolved by caregivers; and (3) no evidence of failure to thrive, fever, or illness [19]. Dyschezia is diagnosed in an infant <9 months of age with ≥10 min of straining and crying before the successful or unsuccessful passage of soft stools without other health problems [19]. Infantile regurgitation is diagnosed in healthy infants 3 weeks to 12 months of age who have regurgitation ≥2 times/day for ≥3 weeks without retching, hematemesis, aspiration, apnea, failure to thrive, feeding or swallowing difficulties, or abnormal posturing [19]. The diagnosis of cyclic vomiting syndrome includes all of the following: (1) ≥2 periods of unremitting paroxysmal vomiting, with or without retching, lasting hours to days within a 6-month period; (2) episodes that are stereotypical in each patient; and (3) episodes separated by weeks to months with return to baseline health between episodes of vomiting [19].

For functional constipation, the child was assessed for successful toilet training. The diagnosis required ≥1 month with ≥2 of the following: (1) ≤2 defecations per week; (2) history of excessive stool retention; (3) history of painful or hard bowel movements; (4) history of large-diameter stools; and (5) presence of a large fecal mass in the rectum. In toilet-trained children, additional criteria included ≥1 episode/week of incontinence and a history of large-diameter stools that may obstruct the toilet [19]. The diagnosis of infant diarrhea required all of the following: (1) daily, painless, recurrent passage of ≥4 large unformed stools; (2) symptoms lasting ≥4 weeks; (3) onset between 6 and 60 months of age; and (4) no failure to thrive if caloric intake is adequate [19].

The duration of exclusive breastfeeding was defined as the period during which the child received only breast milk, either directly or expressed and given via a bottle, with no exposure to formula milk. Exposure to antibiotics and/or antivirals was defined as any use of these medications for a suspected infection.

Data analysis was performed using the R program version 4.2.3 (R Foundation for Statistical Computing, Vienna, Austria). Baseline characteristics are described as median (interquartile range; IQR) or number (percentage). Continuous and categorical data were compared using Fisher’s exact, Mann–Whitney U, or Kruskal–Wallis test, as appropriate. To explore the relationship between potential risk or protective factors and the presence of DGBI, univariate and multivariate logistic regression analyses were performed. Multivariable analyses were performed on the potential factors with a *p*-value <0.2 in univariable logistic regression. The results of logistic regression are presented as ORs with corresponding 95% confidence intervals (CIs). Variance inflation factors were assessed to evaluate multicollinearity in the multivariable models. A *p*-value <0.05 indicated statistical significance. Participants with missing data for key variables were excluded from the respective analyses. The proportion of missing data was assessed, and sensitivity analyses were conducted to ensure the robustness of the findings.

The study was approved by the Ethics Committee of the Faculty of Medicine, Prince of Songkla University (REC 66-317-1-1).

## 3. Results

During the 3-year vaccination follow-up period, 686 children were followed up with until 3 years of age; of these, 568 (80.9%) participated in the DGBI study after their parents provided informed consent. Among the 568 participants (48.6% boys), 139 (24.5%) children experienced symptoms of at least one DGBI.

### 3.1. Early-Life Factors

Of the 568 children, 40.1% (n = 228) were born by cesarean section, and 48.1% and 22.4% were exclusively breastfed for ≥6 months and 1 year, respectively. Of the participating children, 62.3% were exposed to secondhand smoke from family members, and 2.5% had a maternal history of infection requiring antibiotics during pregnancy. Additionally, 482 (84.9%) children received bottle-feeding, with a median initiation age of 1 month (IQR 0–3 months); among these children, 47.9% used the bottles exclusively for expressed breastmilk. Notably, the bottle-feeding group included both infants who received formula and those who were administered expressed breast milk in a bottle.

During the first year of life, 21.1% of children were exposed to at least one course of antibiotics, antiviral drugs, or antifungal drugs. Specifically, 15.8% of children received antibiotics, while 5.5% were first treated with an antiviral drug for influenza or COVID-19. Among the 568 children, 4 had antifungal drugs as their first exposure, and 6 received both antivirals and antibiotics within the first year. By age three, the proportion of children exposed to at least one course of antibiotics, antivirals, or antifungals increased to 78.2%. At 3 years of age, most children consumed three meals per day in addition to 3.5 (range 0–20) ounces of milk on average.

### 3.2. Prevalence of DGBI in Children Aged 3 Years

Regarding the prevalence of DGBI, 24.5% of 3-year-old children had symptoms consistent with at least one DGBI diagnosis. The prevalence of DGBI was significantly higher in girls than in boys (28.1 vs. 20.7%; *p* = 0.041). Among the children studied, one (0.2%) had cyclic vomiting syndrome (CVS), nine (1.6%) had dyschezia, 16 (2.8%) had infantile regurgitation, 26 (4.6%) had infant colic, and 116 (20.4%) had functional constipation. Most children had only one DGBI diagnosis (20.4%), while 3.3, 0.5, and 0.2% had two, three, and five diagnoses, respectively. Of 139 children with at least one DGBI, nine had constipation and history of infant colic, three had constipation and history of infantile regurgitation, two each had constipation with history of dyschezia, infant colic with dyschezia, and dyschezia with infantile regurgitation, one had infant colic with regurgitation, two had constipation with infant colic and regurgitation, one had constipation with infant colic and dyschezia, and one had constipation, infant colic, regurgitation, dyschezia and CVS.

### 3.3. Association Between Early-Life Factors and DGBI Within 3 Years of Age

**Figure 2:**
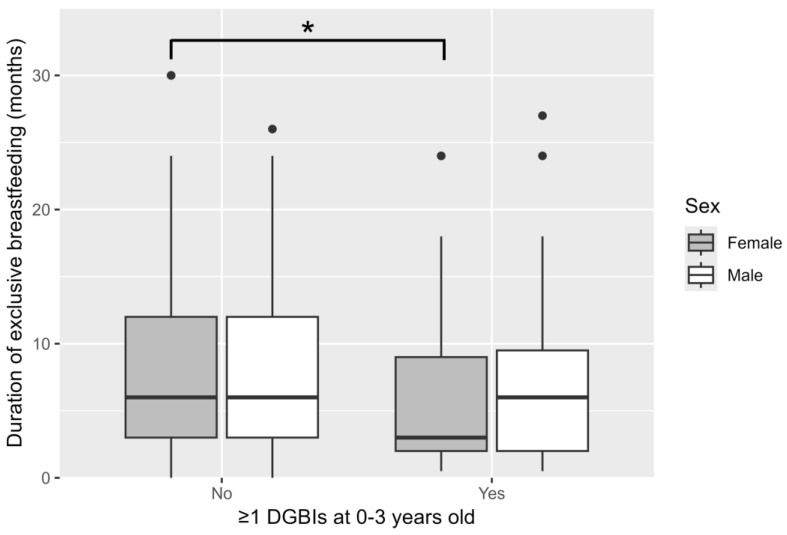
Duration of exclusive breastfeeding in children with and without DGBI. Girls with DGBI stopped being exclusively breastfed earlier than those without DGBI (* *p* = 0.002). DGBIs, Disorders of gut–brain interaction.

Exclusive breastfeeding was stopped earlier in children with DGBI than in those without. Particularly in girls, exclusive breastfeeding ceased at a median age of 3 (IQR 2–9) months in those with DGBI compared to at 6 (IQR 3–12) months in those without DGBI (*p* = 0.002; Figure 2). Children with DGBI were exposed to antibiotics at a later age, whereas they received antivirals earlier than children without DGBI (Table 1). No significant differences in sex, weight, height, mode of delivery, history of food allergy, family history of allergic diseases, or socioeconomic status were identified between children with and without a DGBI diagnosis (Table 1).

**Figure 3:**
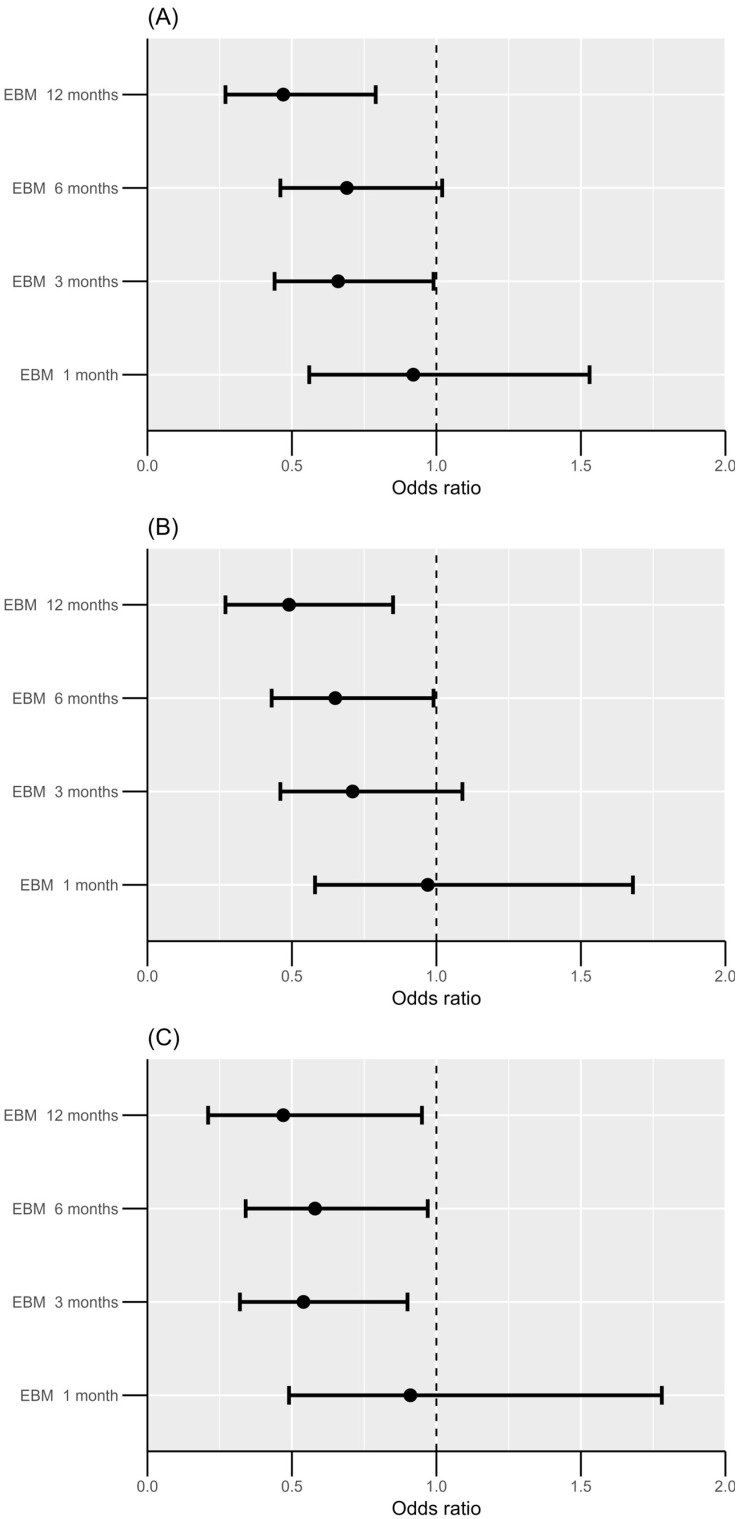
Multivariate analysis showing that exclusive breastfeeding is the most significant protective factor against DGBI (**A**) and constipation (**B**) within 3 years of age. Sensitivity analysis conducted in 69 children who were diagnosed with DGBI (constipation) at 3 years of age showing similar outcomes (**C**). DGBI, Disorders of gut–brain interaction; EBM, Exclusive breastmilk.

After adjusting for potential confounding factors, including exclusive breastfeeding, sex, maternal allergy history, and exposure to secondhand smoking, exposures to antibiotics and antivirals within the first year of life, exclusive breastfeeding was the most significant protective factor against DGBI, particularly if performed for ≥3 months (Table 2, Figure 3A). Exclusive breastfeeding for ≥3 months could reduce the risk of DGBI by 34%, and the protective effect increased to 53% if breastfed for ≥1 year. Moreover, male sex was identified as a protective factor against DGBI (Table 2). Notably, multivariate analysis did not reveal exposure to antibiotics or antivirals as a significant protective or risk factor for DGBI.

### 3.4. Functional Constipation in 3-Year-Old Children

Among the participating children, 116 experienced symptoms consistent with functional constipation, based on ROME IV criteria, within 3 years of age. Among these children, exclusive breastfeeding was stopped at a median age of 4 (IQR 2–8) months in those with DGBI, which was earlier than in those without DGBI [median age of 6 (IQR 3–12) months; *p* = 0.001]. Additionally, children with DGBI were less likely to be toilet-trained than those without DGBI (80.2% vs. 87.4%; *p* = 0.065) (Table 3). However, the success rate of toilet training was not significantly different between the two groups.

Multivariate analysis adjusted for potential factors, including exclusive breastfeeding ≥6 months, sex, exposure to secondhand smoke, toilet training, and exposures to antibiotics and antivirals within the first year of life, was performed. We found that exclusive breastfeeding for ≥6 months was a significant protective factor against functional constipation (OR = 0.65, 95% CI = 0.423–0.99, *p* = 0.047). Additionally, lack of toilet training appeared to increase the risk of developing constipation (OR = 1.65, 95% CI = 0.95–2.82, *p* = 0.070) (Table 4).

Sensitivity analyses were performed by excluding retrospective symptom reports and stratifying the data by sex (Supplementary Tables S1–S3). Among the 69 children diagnosed with functional constipation at 3 years of age, a multivariate analysis was performed by adjusting for exclusive breastfeeding, maternal income, influenza infection, toilet training, and exposure to antibiotics and antivirals during the first year of life. Exclusive breastfeeding for ≥3 months emerged as a significant protective factor against functional constipation (OR = 0.54, 95% CI: 0.32–0.90, *p* = 0.019). Additionally, the absence of toilet training was associated with an increased risk of developing constipation (OR = 1.92, 95% CI: 0.98–3.58, *p* = 0.047) (Figure 3C, Supplementary Table S2). Univariate and multivariate analyses examining the associations between potential factors and each pathological condition included in the DGBI criteria are presented in Supplementary Tables S4–S9 and Supplementary Figure S1.

## 4. Discussion

The concept of early-life programming has been widely accepted as the etiopathogenesis of several diseases, particularly those involved in the gut–brain interaction [2,3,4,5,6,7,8]. The first 3 years of life are critical for postnatal gut and brain development as well as microbial colonization. Within this period, multiple factors or events, such as cesarean section, early-life pain or trauma, early antibiotics use, food allergy, and family stress, can disrupt the bidirectional gut–brain communication and microbiome interaction, leading to DGBI [5,7,10,11,12,13,14,15,16].

In this study, the overall prevalence of DGBI in children aged 3 years was 24.5%, with functional constipation being the most prevalent diagnosis (20.4%). The second-most frequent diagnosis was colic (4.6%) in children <1 year of age, which was retrospectively reported. The overall prevalence of DGBI in our study was similar to that reported in an Italian cohort (19.6–21.1%) [20]. Scarpato et al. also found that the most prevalent DGBI was functional constipation (16.1%) in toddlers (13–48 months) and infant colic (9.3%) among infants (0–12 months) [20]. Compared to Scarpato et al.’s study, our study reported a lower prevalence of infant colic. Given that we interviewed the parents when the child was 3 years old, our findings may have been influenced by recall bias. The prevalence of DGBI in our current study was higher than that reported in a Vietnamese study, which showed that the total prevalence of having at least one DGBI in children aged 0–48 months was 10.0% [18]. This discrepancy may be explained by the different study methods employed (interview vs. questionnaire).

We also demonstrated that exclusive breastfeeding (≥3 months) was the most significant protective factor against DGBI. Notably, there was a sex difference in the prevalence of DGBI in children ≤3 years of age, with a greater prevalence among girls than among boys. Specifically, children with DGBI were exposed to antiviral drugs at an earlier age during their first year of life compared to children without DGBI (8.0 ± 2.4 vs. 10.2 ± 2.0 months; *p* = 0.008). Breast milk contains human breastmilk microbiota and human milk oligosaccharides (HMOs) that promote the production of specific microorganisms; these microorganisms produce metabolites that can modulate gut–brain physiology [2,8,21,22]. A recent in vitro study by Natividad et al. [21] investigated the effect of different HMO on the gut microbiota composition. They collected stool samples from five healthy infants (2–4 months old) and cultivated them together with different groups of HMOs. The HMOs promoted the growth of *Bifidobacterium* and other HMO-consuming bacteria (e.g., *Bacteroides* spp.) and maintained intestinal barrier integrity [21]. Ferrier et al. [22] showed that visceral hypersensitivity-induced mice fed on HMOs had a reduced visceromotor response, as evaluated using colorectal distension, compared with those in the sham group. In line with the findings of Chia et al. [18], we demonstrated that exclusive breastfeeding was the most significant protective factor against DGBI occurrence in children ≤3 years of age. Chia et al. [18] reported that exclusive breastfeeding at 2–3 months and 3–4 months offered protection against infantile regurgitation. However, we found that a longer duration of exclusive breastfeeding was associated with a greater protective effect on DGBI and constipation occurrence (Figure 3). Furthermore, Scarpato et al. revealed that formula feeding was a risk factor for DGBI [20].

Sex differences in DGBI prevalence have been documented in several previous studies [23,24,25]; a majority of these studies examined children aged 4–18 years. We found that among children ≤3 years, male sex was a protective factor against DGBI. According to studies involving animal models, the use of antibiotics in early life could influence the maturation and function of intestinal epithelial cells [26] and disrupt intestinal motility [5]. Additionally, persistent effects on gene expression of intestinal tissue and on gut microbiota diversity were evident [27]. Similar findings were reported in infants receiving antibiotic treatment for suspected early-onset neonatal sepsis; their *Bifidobacterium* spp. abundance was lower than that in healthy controls [28]. Another study by Yassour et al. [4] assessing the gut microbiota progression in children aged 2–36 months showed that gut microbiomes were less stable in those receiving antibiotic treatment than in those not receiving the treatment [4]. In addition, a recent systematic review that evaluated 11 cohort and 11 case–control studies indicated that early antibiotic use could increase the risk of developing GI disorders, including DGBI, in later life [10]. One of the included studies revealed the relationship between neonatal antibiotic use (in the first month of life) and the occurrence of DGBI within the first year of life [29]. However, it remains unclear why, in this study, children with DGBI were exposed to antivirals earlier but received antibiotics at later age compared to those without DGBI. Since bacteria, viruses, and fungi are constituents of the gut microbiota [30,31], viral infections (e.g., influenza, COVID-19) or exposure to antiviral medications may disrupt the gut microbiota [32,33]. Another possibility is that some children were exposed to both antibiotics and antivirals early in life, potentially compounding the impact on gut microbiota development.

Our study failed to identify a significant difference in the prevalence of DGBI between infants born vaginally and those born by cesarean section, despite multiple studies demonstrating the alteration of gut microbiota diversity and *Bacteroides* colonization in infants born by cesarean section [4,6,7], as well as the association with DGBI [29]. Furthermore, early-life stress triggers reactions through the hypothalamic–pituitary–adrenal axis, resulting in intestinal microbiota dysbiosis and abnormal behavioral patterns [15,16]. Limited human studies have examined the connection between DGBI and early-life stress. Another limitation of our study was its inability to determine the effect of family socioeconomic status and history of allergic diseases on the presence of DGBI in children aged 0–3 years. Although the study cohort comprised healthy children enrolled in a vaccine clinical trial, the parents were from diverse socioeconomic backgrounds. However, selection bias was minimized, as all parents received incentives to participate in the clinic visits.

The strength of our study is that we recruited several healthy children and prospectively collected data on their early-life factors from the age of 2 months by interviewing their parents during follow-up visits. Although all illnesses and medication use were reported up to 3 years of age, attention to the existence of GI symptoms was limited unless they were severe. Therefore, the occurrence of DGBI before age 3 may be subject to recall bias, potentially affecting prevalence estimates for each DGBI condition. The involvement of experienced and trained pediatricians in conducting interviews has reduced these risks in our study.

Additional research is necessary to determine the role of gut microbiota and dietary factors in DGBI. A follow-up study should be conducted to prospectively collect data on DGBI symptoms and analyze gut microbiota in this group of participants. Future longitudinal studies assessing the role of early-life factors on the incidence of DGBI during adolescence and adulthood are required.

## 5. Conclusions

We identified a sex difference in the prevalence of DGBI in children ≤ 3 years of age. Breastfeeding is a strong protective factor against DGBI, whereas antibiotic/antiviral exposure may have a negative impact on the prevalence of DGBI, particularly during the first year of life. Allergic diseases, mode of delivery, and socioeconomic status were not associated with the prevalence of DGBI. Hence, exclusive breastfeeding should be continuously promoted, and rational antibiotic/antiviral use should be advocated to prevent the development of DGBI.

## Figures and Tables

**Table 1:** Characteristics of 568 children aged 0–3 years with and without DGBI.

Characteristics	No DGBI (n = 429)	DGBI (n = 139)	*p*-Value
Male sex, n (%)	219 (51.0)	57 (41.0)	0.050
Weight, kg (IQR)	13.4 (12.2–14.7)	13.8 (12.2–15.0)	0.482
Height, cm (IQR)	93.5 (91.0–96.0)	93.0 (91.0–96.0)	0.850
Infection history during pregnancy, n (%)	11 (2.6)	3 (2.2)	1.000
Cesarean section, n (%)	174 (41.0)	54 (38.8)	0.721
Low Apgar score (≤7) at 1 min, n (%)	6 (1.4)	3 (2.2)	0.463
Duration of exclusive breastfeeding, months (IQR)	6.0 (3.0–12.0)	4.0 (2.0–9.0)	0.001
≥3 months of exclusive breastfeeding, n (%)	295 (68.8)	81 (58.3)	0.030
1-year breastfeeding, n (%)	108 (25.2)	19 (13.8)	0.007
Bottle feeding, n (%)	365 (85.1)	117 (84.2)	0.902
Age at first antibiotics/antiviral exposure, months (IQR)	17.0 (11.0–23.0)	18.0 (11.0–24.0)	0.227
Age at first antibiotics/antiviral exposure, n (%)			0.611
No exposure	93 (21.7)	31 (22.3)	
≤6 months	37 (8.6)	7 (5.0)	
>6–12 months	55 (12.8)	21 (15.1)	
>12–24 months	169 (39.4)	52 (37.4)	
>24–36 months	75 (17.5)	28 (20.1)	
Exposure to antibiotics within the 1st year of life, n (%)	75 (17.5)	16 (11.5)	0.125
Age at 1st exposure to antibiotics within the 1st year of life, months *	6.4 ± 2.4	7.8 ± 2.1	0.035
Exposure to antivirals within the 1st year of life, n (%)	20 (4.7)	12 (8.6)	0.120
Age at 1st exposure to antivirals within the 1st year of life, months *	10.2 ± 2.0	8.0 ± 2.4	0.008
Exposure to secondhand smoke, n (%)	260 (60.6)	94 (67.6)	0.166
History of gastroenteritis requiring hospitalization, n (%)	40 (9.3)	14 (10.1)	0.924
Influenza infection within 3 years, n (%)	12 (2.8)	9 (6.5)	0.083
COVID-19 infection within 1st 6-month, n (%)	0 (0)	2 (1.4)	0.060
History of food allergy, n (%)	29 (7.4)	8 (6.3)	0.831
Asthma, n (%)	11 (2.8)	2 (1.6)	0.744
Atopic dermatitis, n (%)	24 (6.1)	7 (5.5)	0.960
Family history of allergic diseases			
- Father, n (%)	56 (13.1)	19 (13.7)	0.966
- Mother, n (%)	50 (11.7)	23 (16.5)	0.176
- Sibling, n (%)	173 (40.3)	60 (43.2)	0.623
Socioeconomic status			
- Paternal income, Baht/month (IQR)	15,000 (9500–20,000)	15,000 (9000–20,000)	0.906
- Maternal income, Baht/month (IQR)	9000 (0–15,000)	10,000 (0–15,000)	0.867
Paternal education, n (%)			0.807
- Bachelor’s degree	98 (23.5)	33 (24.4)	
- Higher than bachelor’s degree	8 (1.9)	2 (1.5)	
- Less than or equal to high school	235 (56.4)	71 (52.6)	
- Vocational training	76 (18.2)	29 (21.5)	
Maternal education, n (%)			0.700
- Bachelor’s degree	185 (43.1)	53 (38.1)	
- Higher than bachelor’s degree	13 (3.0)	4 (2.9)	
- Less than or equal to high school	173 (40.3)	59 (42.4)	
- Vocational training	58 (13.5)	23 (16.5)	

* Mean ± standard deviation; IQR, interquartile range; DGBI, Disorders of gut–brain interaction.

**Table 2:** Multivariate analysis showing factors associated with the prevalence of DGBI in 3-year-old children (n = 566).

Factor	Crude OR (95% CI)	Adj. OR (95% CI)	*p*-Value (Wald’s Test)	*p*-Value (LR-Test)
Exclusive breastfeeding for ≥3 months: Yes vs. No	0.63 (0.43, 0.94)	0.66 (0.44, 0.99)	0.043	0.044
Sex: Male vs. Female	0.67 (0.45, 0.98)	0.66 (0.44, 0.97)	0.038	0.037
Maternal history of allergy: Yes vs. No	1.50 (0.87, 2.54)	1.65 (0.94, 2.83)	0.073	0.080
Exposure to secondhand smoke: Yes vs. No	1.36 (0.91, 2.05)	1.29 (0.86, 1.96)	0.231	0.228
Exposure to antibiotics within 1 year: Yes vs. No	0.61 (0.33, 1.07)	0.60 (0.32, 1.05)	0.086	0.074
Exposure to antivirals within 1 year: Yes vs. No	1.93 (0.89, 4.01)	2.02 (0.92, 4.30)	0.071	0.079

OR, odds ratio; CI, confidence interval; Adj., adjusted; DGBI, disorders of gut–brain interaction; LR, likelihood ratio.

**Table 3:** Characteristics of 568 children with and without constipation.

Characteristics	No Constipation (n = 437)	Constipation (n = 116)	*p*-Value
Male sex, n (%)	228 (50.4)	48 (41.4)	0.101
Weight, kg (IQR)	13.4 (12.2–14.7)	13.8 (12.3–15.0)	0.414
Height, cm (IQR)	93.5 (91.0–96.0)	93.0 (91.0–96.0)	0.918
Infection history during pregnancy, n (%)	13 (2.9)	1 (0.9)	0.320
Cesarean section, n (%)	183 (40.9)	45 (38.8)	0.754
Low Apgar score (≤7) at 1 min, n (%)	8 (1.8)	1 (0.9)	0.694
Duration of exclusive breastfeeding, months (IQR)	6.0 (3.0–12.0)	4.0 (2.0–8.0)	0.001
≥6 months of exclusive breastfeeding, n (%)	228 (50.4)	45 (38.8)	0.033
1-year breastfeeding, n (%)	111 (24.7)	16 (13.8)	0.017
Bottle feeding, n (%)	383 (84.7)	99 (85.3)	0.985
Age at first antibiotics/antiviral exposure, months (IQR)	17.0 (11.0–23.0)	18.0 (11.8–23.2)	0.271
Days of antibiotics/antiviral exposure within 6 months, days (IQR)	7.0 (3.0–10.0)	7.0 (5.0–11.0)	0.494
Days of antibiotics/antiviral exposure within 12 months, days (IQR)	7.0 (5.0–10.0)	5.0 (5.0–10.0)	0.281
Exposure to antibiotics within the 1st year of life, n (%)	77 (17.0)	14 (12.1)	0.246
Age at 1st exposure to antibiotics within the 1st year of life, months *	6.4 ± 2.4	7.8 ± 2.3	0.040
Exposure to antivirals within the 1st year of life, n (%)	25 (5.5)	7 (6.0)	1.000
Age at 1st exposure to antivirals within the 1st year of life, months *	10.0 ± 2.0	7.2 ± 2.5	0.004
Exposure to secondhand smoke, n (%)	275 (60.8)	79 (68.1)	0.183
Influenza infection within 3 years, n (%)	13 (2.9)	8 (6.9)	0.053
COVID-19 infection within 1st 6-month, n (%)	0	2 (1.7)	0.041
COVID-19 infection within 1st year, n (%)	25 (5.5)	7 (6.0)	1.000
Toilet training, n (%)	395 (87.4)	93 (80.2)	0.065
Successful toilet training, n (%)	333 (84.3)	74 (80.4)	0.456
History of food allergy, n (%)	33 (8.0)	4 (3.8)	0.207
Asthma, n (%)	12 (2.9)	1 (1.0)	0.482
Atopic dermatitis, n (%)	26 (6.3)	5 (4.7)	0.710
Family history of allergic diseases			
- Father, n (%)	61 (13.5)	14 (12.1)	0.802
- Mother, n (%)	54 (11.9)	19 (16.4)	0.264
- Sibling, n (%)	183 (40.5)	50 (43.1)	0.685
Socioeconomic status			
- Paternal income, Baht/month (IQR)	15,000 (9500–20,000)	14,000 (9000–20,000)	0.857
- Maternal income, Baht/month (IQR)	9000 (0–15,000)	10,000 (0–15,000)	0.689
Paternal education, n (%)			0.608
- Bachelor’s degree	102 (23.2)	29 (25.9)	
- Higher than bachelor’s degree	8 (1.8)	2 (1.8)	
- Less than or equal to high school	250 (56.8)	56 (50.0)	
- Vocational training	80 (18.2)	25 (22.3)	
Maternal education, n (%)			0.988
- Bachelor’s degree	190 (42.0)	48 (41.4)	
- Higher than bachelor’s degree	13 (2.9)	4 (3.4)	
- Less than or equal to high school	185 (40.9)	47 (40.5)	
- Vocational training	64 (14.2)	17 (14.7)	

* Mean ± standard deviation; IQR, interquartile range.

**Table 4:** Multivariate analysis showing factors associated with the prevalence of constipation in 3-year-old children (n = 566).

Factor	Crude OR (95% CI)	Adj. OR (95% CI)	*p*-Value (Wald’s Test)	*p*-Value (LR-Test)
Exclusive breastfeeding for ≥6 months: Yes vs. No	0.62 (0.41, 0.94)	0.65 (0.43, 0.99)	0.047	0.046
Sex: Male vs. Female	0.69 (0.46, 1.05)	0.69 (0.45, 1.05)	0.085	0.083
Exposure to secondhand smoke: Yes vs. No	1.37 (0.90, 2.14)	1.28 (0.83, 2.00)	0.277	0.273
Lack of toilet training: Yes vs. No	1.71 (0.99, 2.89)	1.65 (0.95, 2.82)	0.070	0.077
Exposure to antibiotics within 1 year: Yes vs. No	0.67 (0.35, 1.20)	0.68 (0.35, 1.22)	0.213	0.199
Exposure to antivirals within 1 year: Yes vs. No	1.10 (0.43, 2.48)	1.13 (0.44, 2.59)	0.787	0.789

OR, odds ratio; CI, confidence interval; Adj., adjusted; DGBI, disorders of gut–brain interaction; LR, likelihood ratio.

## Data Availability

Data supporting the findings of this study are provided in the Supplementary File and are available from the corresponding author upon reasonable request.

## Supplementary Materials

The following supporting information can be downloaded at: https://www.mdpi.com/article/10.3390/children12111430/s1.

## Abbreviations

The following abbreviations are used in this manuscript:

CI	Confidence interval
DGBI	Disorders of gut–brain interaction
GI	Gastrointestinal
HMO	Human milk oligosaccharides
IQR	Interquartile range
OR	Odds ratio
