# Supplementary material for: Early-Life Events and the Prevalence of Gut–Brain Interaction Disorders in Children
Source: Children (Basel). 2025 Oct 23;12(11):1430. doi: 10.3390/children12111430 (PMC12651323; doi:10.3390/children12111430)
Supplement: Supplementary file 1 [file children-12-01430-s001.zip › children-3905728 supplementary tables.pdf]

Table S1. Characteristics of 69 children with constipation at 3 years of age, compared to those without constipation (excluding retrospective symptom reports).

Characteristics	No constipation (n=499)	Constipation (n=69)	*P* value
Male gender, n (%)	243 (48.7)	33 (47.8)	0.994
Weight, kg (IQR)	13.4 (12.2-14.8)	13.8 (12.2-15.0)	0.400
Height, cm (IQR)	93.0 (91.0-96.0)	94.0 (91.0-96.0)	0.488
History of infection during pregnancy, n (%)	13 (2.6)	1 (1.4)	1.000
Cesarean section, n (%)	199 (40.3)	29 (42.0)	0.884
Low Apgar score (≤7) at 1 min, n (%)	8 (1.6)	1 (1.4)	1.000
Duration of exclusive breastfeeding, months (IQR)	6.0 (3.0-12.0)	3.0 (2.0-8.0)	0.002
≥3-month-exclusive breastfeeding, n (%)	339 (67.9)	37 (53.6)	0.026
Bottle feeding, n (%)	424 (85.0)	58 (84.1)	0.985
Age at first exposure to antibiotics/antivirus, months (IQR)	17.0 (11.0-23.0)	14.5 (8.0-22.0)	0.165
Days of antibiotics/antiviral exposure within 6 months, days (IQR)	7.0 (3.0-10.0)	6.0 (5.0-9.2)	0.892
Days of antibiotics/antiviral exposure within 12 months, days (IQR)	7.0 (5.0-10.0)	5.0 (4.5-7.0)	0.157
Exposure to antibiotics within the 1st year of life, n (%)	79 (15.8)	11 (15.9)	1.000
Age at 1st antibiotics exposure within the 1st year of life, months (mean±SD)	6.4±2.4	7.5±2.5	0.072
Exposure to antivirals within the 1st year of life, n (%)	25 (5.0)	6 (8.7)	0.251
Age at 1st antivirals exposure within the 1st year of life, months (mean±SD)	10.1±1.9	7.0±2.7	0.003
Influenza infection within 3 years, n (%)	16 (3.2)	5 (7.2)	0.161
COVID-19 infection within 1st year, n (%)	26 (5.2)	4 (5.8)	0.775
Toilet training, n (%)	434 (87.0)	54 (78.3)	0.077

Successful toilet training, n (%)	367 (84.6)	40 (75.5)	0.136
Exposure of smoking, n (%)	309 (61.9)	45 (65.2)	0.692
History of food allergy, n (%)	36 (7.9)	1 (1.6)	0.070
Asthma, n (%)	13 (2.8)	0	0.383
Atopic dermatitis, n (%)	28 (6.1)	3 (4.8)	1.000
Family history of allergic diseases			
- Father, n (%)	65 (13.0)	10 (14.5)	0.883
- Mother, n (%)	64 (12.8)	9 (13.0)	1.000
- Sibling, n (%)	201 (40.3)	32 (46.4)	0.404
Socioeconomic status			
- Paternal income, Baht/month (IQR)	15000 (9500-20000)	12000 (9000-20000)	0.787
- Maternal income, Baht/month (IQR)	9000 (0-15000)	10000 (6000-15000)	0.149
Paternal education, n (%)			0.472
- Bachelor's degree	113 (23.3)	18 (27.3)	
- Higher than bachelor's degree	8 (1.6)	2 (3.0)	
- Less than or equal to high school	275 (56.6)	31 (47.0)	
- Vocational tract	90 (18.5)	25 (22.7)	
Maternal education, n (%)			0.489
- Bachelor's degree	212 (42.5)	26 (37.7)	
- Higher than bachelor's degree	13 (2.6)	4 (5.8)	
- Less than or equal to high school	203 (40.7)	29 (42.0)	
- Vocational tract	71 (14.2)	10 (14.5)	

IQR, interquartile range.

Table S2. Multivariate analysis of potential factors for having constipation at 3 years of age (excluding retrospective symptom reports, n=564).

Characteristics	Crude OR (95%CI)	Adj. OR (95%CI)	P-value (Wald's test)	P-value (LR-test)
Exclusive breastfeeding for ≥3 months: Yes vs No	0.54 (0.33,0.91)	0.54 (0.32,0.90)	0.018	0.019
Maternal income	1.00 (1.00,1.00)	1.00 (1.00,1.00)	0.779	0.781
Influenza infection: Yes vs No	2.35 (0.75,6.23)	2.40 (0.72,6.77)	0.117	0.141
Lack of toilet training: Yes vs No	1.85 (0.96,3.39)	1.91 (0.98,3.57)	0.048	0.057
Exposure to antibiotics within 1 year: Yes vs No	1.00 (0.48,1.93)	1.03 (0.49,2.00)	0.941	0.941
Exposure to antivirals within 1 year: Yes vs No	1.73 (0.62,4.10)	1.42 (0.49,3.55)	0.480	0.493

Table S3. Characteristics of 139 children with DGBI within 3 years of age, compared by sex.

Characteristics	Female (n=82)	Male (n=57)	*P* value
Weight, kg (IQR)	13.2 (12.1-15.0)	13.8 (12.7-15.0)	0.161
Height, cm (mean±SD)	93.0±3.8	94.0±3.5	0.119
DGBI diagnosis within 3 years of age, n (%)			
- infant colic	18 (22.0)	8 (14.0)	0.339
- infantile regurgitation	10 (12.2)	6 (10.5)	0.974
- dyschezia	7 (8.5)	2 (3.5)	0.308
- cyclic vomiting syndrome	1 (1.2)	0	1.000
- functional constipation	68 (82.9)	48 (84.2)	1.000
Overall DGBI within 1 year of age, n (%)	30 (36.6)	16 (28.1)	0.386
DGBI (constipation) diagnosed at 3 years of age, n (%)	36 (43.9)	33 (57.9)	0.147
History of infection during pregnancy, n (%)	1 (1.2)	2 (3.5)	0.568
Cesarean section, n (%)	27 (32.9)	27 (47.4)	0.123
Low Apgar score (≤7) at 1 min, n (%)	2 (2.4)	1 (1.8)	1.000
Duration of exclusive breastfeeding, months (IQR)	3.0 (2.0-9.0)	6.0 (2.0-9.5)	0.361
≥6-month-exclusive breastfeeding, n (%)	29 (35.4)	27 (47.4)	0.214

Bottle feeding, n (%)	70 (85.4)	47 (82.5)	0.821
Exposure to antibiotics/antivirals within 6 months-1st year of life, n (%)	8 (9.8)	13 (22.8)	0.061
Age at first exposure to antibiotics/antivirals, months (mean±SD)	18.3±7.8	17.5±9.3	0.634
Days of antibiotics/antiviral exposure within 6 months, days (mean±SD)	8.6±4.4	6.0±1.4	NA
Days of antibiotics/antiviral exposure within 12 months, days (IQR)	7.0 (5.0-7.8)	5.0 (5.0-8.5)	0.468
Exposure to antibiotics within the 1st year of life, n (%)	9 (11.0)	7 (12.3)	1.000
Age at 1st antibiotics exposure within the 1st year of life, months (mean±SD)	7.0±1.9	8.7±2.2	0.120
Exposure to antivirals within the 1st year of life, n (%)	3 (3.7)	9 (15.8)	0.016
Age at 1st antivirals exposure within the 1st year of life, months (mean±SD)	7.9±4.4	8.0±1.8	0.929
Influenza infection within 3 years, n (%)	3 (3.7)	6 (10.5)	0.160
COVID-19 infection within 1st year, n (%)	4 (4.9)	7 (12.3)	0.125
COVID-19 infection within 3 years, n (%)	19 (23.2)	15 (26.3)	0.823
Toilet training, n (%)	70 (85.4)	44 (77.2)	0.313
Successful toilet training, n (%)	60 (87.0)	35 (79.5)	0.432
Exposure of smoking, n (%)	60 (73.2)	34 (59.6)	0.136
History of food allergy, n (%)	4 (5.3)	4 (7.7)	0.715
Asthma, n (%)	0	2 (3.8)	0.166
Atopic dermatitis, n (%)	2 (2.6)	5 (9.6)	0.119
Family history of allergic diseases			
- Father, n (%)	12 (14.6)	7 (12.3)	0.884
- Mother, n (%)	12 (14.6)	11 (19.3)	0.620
- Sibling, n (%)	37 (45.1)	23 (40.4)	0.701

Socioeconomic status			
- Paternal income, Baht/month (IQR)	15000 (9500-20000)	12000 (10000-17750)	0.689
- Maternal income, Baht/month (IQR)	10000 (0-15000)	9000 (0-12000)	0.338
Paternal education, n (%)			0.798
- Bachelor's degree	20 (25)	13 (23.6)	
- Higher than bachelor's degree	1 (1.2)	1 (1.8)	
- Less than or equal to high school	44 (55.0)	27 (49.1)	
- Vocational tract	15 (18.8)	14 (25.5)	
Maternal education, n (%)			0.371
- Bachelor's degree	28 (34.1)	25 (43.9)	
- Higher than bachelor's degree	2 (2.4)	2 (3.5)	
- Less than or equal to high school	35 (42.7)	24 (42.1)	
- Vocational tract	17 (20.7)	6 (10.5)	

IQR, interquartile range

Table S4. Characteristics of 26 children with infant colic at 1 year of age, compared to those without colic (retrospective symptom reports).

**Characteristics**	**No colic (n=542)**	**Infant colic (n=26)**	***P* value**
Male gender, n (%)	268 (49.4)	8 (30.8)	0.097
Weight, kg (IQR)	13.4 (12.2-14.8)	14.1 (12.3-16.1)	0.112
Height, cm (IQR)	93.5 (91.0-96.0)	92.5 (90.2-96.8)	0.781
History of infection during pregnancy, n (%)	13 (2.4)	1 (3.8)	0.485
Cesarean section, n (%)	218 (40.6)	10 (38.5)	0.990
Low Apgar score (≤7) at 1 min, n (%)	7 (1.3)	2 (7.7)	0.060
Duration of exclusive breastfeeding, months (IQR)	6.0 (3.0-12.0)	5.0 (2.0-12.0)	0.999
≥3-month-exclusive breastfeeding, n (%)	362 (66.8)	14 (53.8)	0.250
Bottle feeding, n (%)	462 (85.2)	20 (76.9)	0.260

Age at first exposure to antibiotics/antivirus, months (IQR)	17.0 (11.0-23.0)	17 (9.5-26.2)	0.716
Days of antibiotics/antiviral exposure within 12 months, days (IQR)	7.0 (5.0-10.0)	7.0 (5.0-8.5)	0.813
Exposure to antibiotics within the 1st year of life, n (%)	87 (16.1)	4 (15.4)	1.000
Age at 1st antibiotics exposure within the 1st year of life, months (mean±SD)	6.5±2.4	8.1±2.5	0.194
Exposure to antivirals within the 1st year of life, n (%)	27 (5.0)	5 (19.2)	0.012
Age at 1st antivirals exposure within the 1st year of life, months (IQR)	10.7 (9.0-11.5)	7.2 (6.7-7.5)	0.019
Influenza infection within 3 years, n (%)	18 (3.3)	3 (11.5)	0.066
COVID-19 infection within 1st year, n (%)	28 (5.2)	4 (15.4)	0.052
Exposure of smoking, n (%)	336 (62.0)	18 (69.2)	0.591
History of food allergy, n (%)	36 (7.3)	1 (4.2)	1.000
Atopic dermatitis, n (%)	31 (6.2)	0	0.387
Family history of allergic diseases			
- Father, n (%)	72 (13.3)	3 (11.5)	1.000
- Mother, n (%)	70 (12.9)	3 (11.5)	1.000
- Sibling, n (%)	225 (41.5)	8 (30.8)	0.377
Socioeconomic status			
- Paternal income, Baht/month (IQR)	15000 (9500-20000)	15000 (9000-18000)	0.785
- Maternal income, Baht/month (IQR)	9000 (0-15000)	9750 (0-15000)	0.994
Paternal education, n (%)			0.887
- Bachelor's degree	126 (23.9)	5 (20.0)	
- Higher than bachelor's degree	10 (1.9)	0	
- Less than or equal to high school	290 (55.0)	16 (64.0)	
- Vocational tract	101 (19.2)	4 (16.0)	

Maternal education, n (%)			0.669
- Bachelor's degree	229 (42.3)	9 (34.6)	
- Higher than bachelor's degree	17 (3.1)	0	
- Less than or equal to high school	220 (40.6)	12 (46.2)	
- Vocational tract	76 (14.0)	5 (19.2)	

IQR, interquartile range

Table S5. Multivariate analysis of potential factors for infant colic at 1 year of age (retrospective symptom reports, n=566).

Characteristics	Crude OR (95%CI)	Adj. OR (95%CI)	P-value (Wald's test)	P-value (LR-test)
Exclusive breastfeeding for ≥3 months: Yes vs No	0.58 (0.26,1.30)	0.72 (0.31,1.67)	0.427	0.431
Sex: Male vs Female	0.45 (0.18,1.03)	0.41 (0.16,0.94)	0.044	0.036
Weight, kg	1.10 (0.94,1.25)	1.12 (0.96,1.29)	0.130	0.150
Asphyxia: Yes vs No	6.37 (0.92,28.08)	5.28 (0.63,28.88)	0.078	0.114
Exposure to antibiotics within 1 year: Yes vs No	0.95 (0.27,2.56)	0.99 (0.28,2.77)	0.998	0.998
Exposure to antivirals within 1 year: Yes vs No	4.54 (1.43,12.18)	3.96 (1.15,11.41)	0.016	0.031

Table S6. Characteristics of 16 children with infantile regurgitation at 1 year of age, compared to those without regurgitation (retrospective symptom reports).

Characteristics	No regurgitation (n=552)	Infantile regurgitation (n=16)	*P* value
Male gender, n (%)	270 (48.9)	6 (37.5)	0.518
Weight, kg (IQR)	13.4 (12.2-14.9)	13.7 (12.6-14.6)	0.817
Height, cm (IQR)	93.5 (91.0-96.0)	93.0 (91.8-95.2)	0.798
History of infection during pregnancy, n (%)	13 (2.4)	1 (6.2)	0.333
Cesarean section, n (%)	224 (41)	4 (25)	0.306
Low Apgar score (≤7) at 1 min, n (%)	8 (1.4)	1 (6.2)	0.228
Duration of exclusive breastfeeding, months (IQR)	6.0 (3.0-12.0)	3.0 (1.8-6.5)	0.066
≥3-month-exclusive breastfeeding, n (%)	368 (66.7)	8 (50.0)	0.262

Bottle feeding, n (%)	467 (84.6)	15 (93.8)	0.487
Age at first exposure to antibiotics/antivirus, months (IQR)	17.0 (11.0-23.0)	23 (9.0-28.0)	0.274
Days of antibiotics/antiviral exposure within 12 months, days (IQR)	7.0 (5.0-10.0)	7.0 (5.0-7.0)	0.596
Age at 1st antibiotics exposure within the 1st year of life, months (range)	6.6 (1.9-11.6)	8.4 (7.9-8.8)	NA
Exposure to antibiotics within the 1st year of life, n (%)	89 (16.1)	2 (12.5)	1.000
Exposure to antivirals within the 1st year of life, n (%)	29 (5.3)	3 (18.8)	0.055
Age at 1st antivirals exposure within the 1st year of life, months (IQR)	10.2 (8.0-11.3)	9.5 (8.5-10.7)	0.897
Influenza infection within 3 years, n (%)	21 (3.8)	0	1.000
COVID-19 infection within 1st year, n (%)	29 (5.3)	3 (18.8)	0.055
Exposure of smoking, n (%)	342 (62.0)	12 (75)	0.424
History of food allergy, n (%)	34 (6.7)	3 (21.4)	0.070
Atopic dermatitis, n (%)	29 (5.7)	2 (14.3)	0.200
Family history of allergic diseases			
- Father, n (%)	71 (12.9)	4 (25.0)	0.249
- Mother, n (%)	71 (12.9)	2 (12.5)	1.000
- Sibling, n (%)	227 (41.1)	6 (37.5)	0.974
Socioeconomic status			
- Paternal income, Baht/month (IQR)	15000 (9500-20000)	15000 (9500-16250)	0.643
- Maternal income, Baht/month (IQR)	9000 (0-15000)	9500 (0-11250)	0.611
Paternal education, n (%)			0.052
- Bachelor’s degree	130 (24.3)	1 (6.2)	
- Higher than bachelor’s degree	9 (1.7)	1 (6.2)	
- Less than or equal to high school	293 (54.7)	13 (81.2)	

- Vocational tract	104 (19.4)	1 (6.2)	
Maternal education, n (%)			0.099
- Bachelor's degree	235 (42.6)	3 (18.8)	
- Higher than bachelor's degree	17 (3.1)	0	
- Less than or equal to high school	224 (40.6)	8 (50.0)	
- Vocational tract	76 (13.8)	5 (31.2)	

IQR, interquartile range.

Table S7. Multivariate analysis of potential factors for infantile regurgitation at 1 year of age (retrospective symptom reports, n=550).

**Characteristics**	**Crude OR (95%CI)**	**Adj. OR (95%CI)**	**P-value (Wald's test)**	**P-value (LR-test)**
Exclusive breastfeeding for ≥3 months: Yes vs No	0.48 (0.18,1.34)	0.53 (0.18,1.51)	0.226	0.229
Exposure to antibiotics within 1 year: Yes vs No	0.76 (0.12,2.78)	0.73 (0.11,2.88)	0.695	0.686
Exposure to antivirals within 1 year: Yes vs No	4.19 (0.92,13.92)	4.08 (0.85,14.93)	0.046	0.074
Paternal education: ref.= Bachelor's degree				0.119
- Higher than bachelor's degree	14.44 (0.54,386.43)	21.02 (0.74,608.34)	0.043	
- Less than or equal to high school	5.77 (1.13,105.33)	2.99 (0.49,58.33)	0.322	
- Vocational tract	1.25 (0.05,31.86)	0.73 (0.03,19.62)	0.827	
Maternal education: ref.= Bachelor's degree				0.136
- Higher than bachelor's degree	0 (NA,1.433e+22)	0 (0,3.387e+27)	0.992	
- Less than or equal to high school	2.89 (0.82,13.33)	2.38 (0.57,13.93)	0.274	
- Vocational tract	5.30 (1.27,26.33)	6.05 (1.24,38.46)	0.034	

Table S8. Characteristics of 9 children with dyschezia at 1 year of age, compared to those without dyschezia (retrospective symptom reports).

**Characteristics**	**No dyschezia (n=559)**	**Dyschezia (n=9)**	***P* value**
Male gender, n (%)	274 (49.0)	2 (22.2)	0.178
Weight, kg (IQR)	13.5 (12.2-14.9)	14.2 (12.2-14.4)	0.984
Height, cm (IQR)	93.5 (91.0-96.0)	93.0 (92.0-95.0)	0.895
History of infection during pregnancy, n (%)	12 (2.1)	2 (22.2)	0.018
Cesarean section, n (%)	224 (40.4)	4 (44.4)	1.000

Low Apgar score (≤7) at 1 min, n (%)	9 (1.6)	0	1.000
Duration of exclusive breastfeeding, months	6.0 (3.0-12.0)	3.0 (2.0-6.8)	0.247
≥3-month-exclusive breastfeeding, n (%)	372 (66.5)	4 (44.4)	0.174
Bottle feeding, n (%)	474 (84.8)	8 (88.9)	1.000
Age at first exposure to antibiotics/antivirus, months (IQR)	17.0 (11.0-23.0)	20.5 (8.5-29.8)	0.459
Days of antibiotics/antiviral exposure within 12 months, days (IQR)	7.0 (5.0-10.0)	7.0 (7.0-7.0)	0.964
Exposure to antibiotics within the 1st year of life, n (%)	88 (15.7)	3 (33.3)	0.162
Age at 1st antibiotics exposure within the 1st year of life, months (range)	6.4 (1.9-11.6)	7.9 (6.7-9.6)	NA
Exposure to antivirals within the 1st year of life, n (%)	31 (5.5)	1 (11.1)	0.409
Age at 1st antivirals exposure within the 1st year of life, months (mean±SD)	6.6±2.4	8.1±1.5	0.284
Influenza infection within 3 years, n (%)	20 (3.6)	1 (11.1)	0.290
COVID-19 infection within 1st year, n (%)	32 (5.7)	0	1.000
Exposure of smoking, n (%)	349 (62.4)	5 (55.6)	0.735
History of food allergy, n (%)	37 (7.2)	0	1.000
Atopic dermatitis, n (%)	31 (6.0)	0	1.000
Family history of allergic diseases			
- Father, n (%)	74 (13.2)	1 (11.1)	1.000
- Mother, n (%)	72 (12.9)	1 (11.1)	1.000
- Sibling, n (%)	228 (40.8)	5 (55.6)	0.498
Socioeconomic status			
- Paternal income, Baht/month (IQR)	15000 (9500-20000)	10000 (9000-12000)	0.261
- Maternal income, Baht/month (IQR)	9000 (0-15000)	9000 (0-10000)	0.455
Paternal education, n (%)			0.923

- Bachelor's degree	129 (23.8)	2 (22.2)	
- Higher than bachelor's degree	10 (1.8)	0	
- Less than or equal to high school	300 (55.2)	6 (66.7)	
- Vocational tract	104 (19.2)	1 (11.1)	
Maternal education, n (%)			0.323
- Bachelor's degree	236 (42.2)	2 (22.2)	
- Higher than bachelor's degree	17 (3.0)	0	
- Less than or equal to high school	228 (40.8)	4 (44.4)	
- Vocational tract	78 (14.0)	3 (33.3)	

IQR, interquartile range

Table S9. Multivariate analysis of potential factors for dyschezia at 1 year of age (retrospective symptom reports, n=566).

Characteristics	Crude OR (95%CI)	Adj. OR (95%CI)	P-value (Wald's test)	P-value (LR-test)
Exclusive breastfeeding for ≥3 months: Yes vs No	0.40 (0.10,1.54)	0.50 (0.12,2.07)	0.326	0.327
Sex: Male vs Female	0.30 (0.04,1.24)	0.27 (0.04,1.17)	0.113	0.082
Exposure to antibiotics within 1 year: Yes vs No	2.68 (0.56,10.34)	2.83 (0.56,11.58)	0.161	0.187
Maternal infection history during pregnancy: Yes vs No	13.02 (1.82,61.12)	11.03 (1.41,59.36)	0.008	0.026
